# Tracheobronchial Foreign Body Aspiration: Spontaneous Expectoration After Beta-2 Agonist Administration

**DOI:** 10.7759/cureus.68804

**Published:** 2024-09-06

**Authors:** Abdelkrim Khannous, Adraa Khannous, Salma Abrar, Selma Abdala, Hind Serhane

**Affiliations:** 1 Pulmonology Department, Hassan II Regional Hospital, Souss-Massa University Hospital, LARISS Laboratory, Faculty of Medicine and Pharmacy of Agadir, Ibn Zohr University, Agadir, MAR; 2 Pulmonology Department, Sidi Mohamed Ben Abdellah University, Fès, MAR

**Keywords:** chest x ray, hijab pin, salbutamol, spontaneous foreign body expectoration, tracheobronchial foreign body aspiration

## Abstract

Tracheobronchial foreign body (TFB) aspiration in adults is uncommon but can be life-threatening, often due to differences in airway sizes and reflexes. Symptoms associated with TFB are typically choking episodes followed by cough and dyspnea, but sometimes it can lead to acute asphyxiation.

Chest radiography and computed tomography can provide information about the foreign body, its characteristics, and its location, however, bronchoscopy remains the preferred method for diagnosis and removal.

In this case report, we describe an instance of hijab pin aspiration where the patient expectorated the foreign body immediately following the administration of inhaled salbutamol.

## Introduction

Tracheobronchial foreign body (TFB) aspiration in adults is a rare but serious condition that can be life-threatening. The clinical presentation can vary widely, ranging from the typical sudden onset of a choking event followed by a persistent cough to more insidious symptoms that mimic asthma, tuberculosis, or unresolved pneumonia [1].

In adults, the risk factors for TFB aspiration include an altered mental status, a low level of consciousness from trauma, neurological disease resulting in impaired airway reflexes, and dental procedures [2]. Imaging exams like chest radiography and CT scans provide valuable information about the size, location, and characteristics of the foreign body, and flexible bronchoscopy remains the preferred method for diagnosing and managing TFB aspiration.

In some rare cases, we can assist in a spontaneous expectoration of TFB after administration of beta-2 agonist. Herein, we report a case of a young woman with TFB aspiration who expectorated a hijab pin immediately after taking inhaled salbutamol.

## Case presentation

A 34-year-old woman with a prior history of asthma under treatment (fluticasone propionate/salmeterol diskus inhalation powder 250/50 mcg, one inhalation twice daily + as-needed salbutamol inhaler) presented to the emergency department with sudden-onset choking episode, followed by cough, wheezing, and shortness of breath. The patient reported that while she was wearing her hijab, she had accidentally aspirated her hijab pin used to secure the fabric. Physical examination revealed decreased air entry and wheezing on the left lung side.

**Figure 1:**
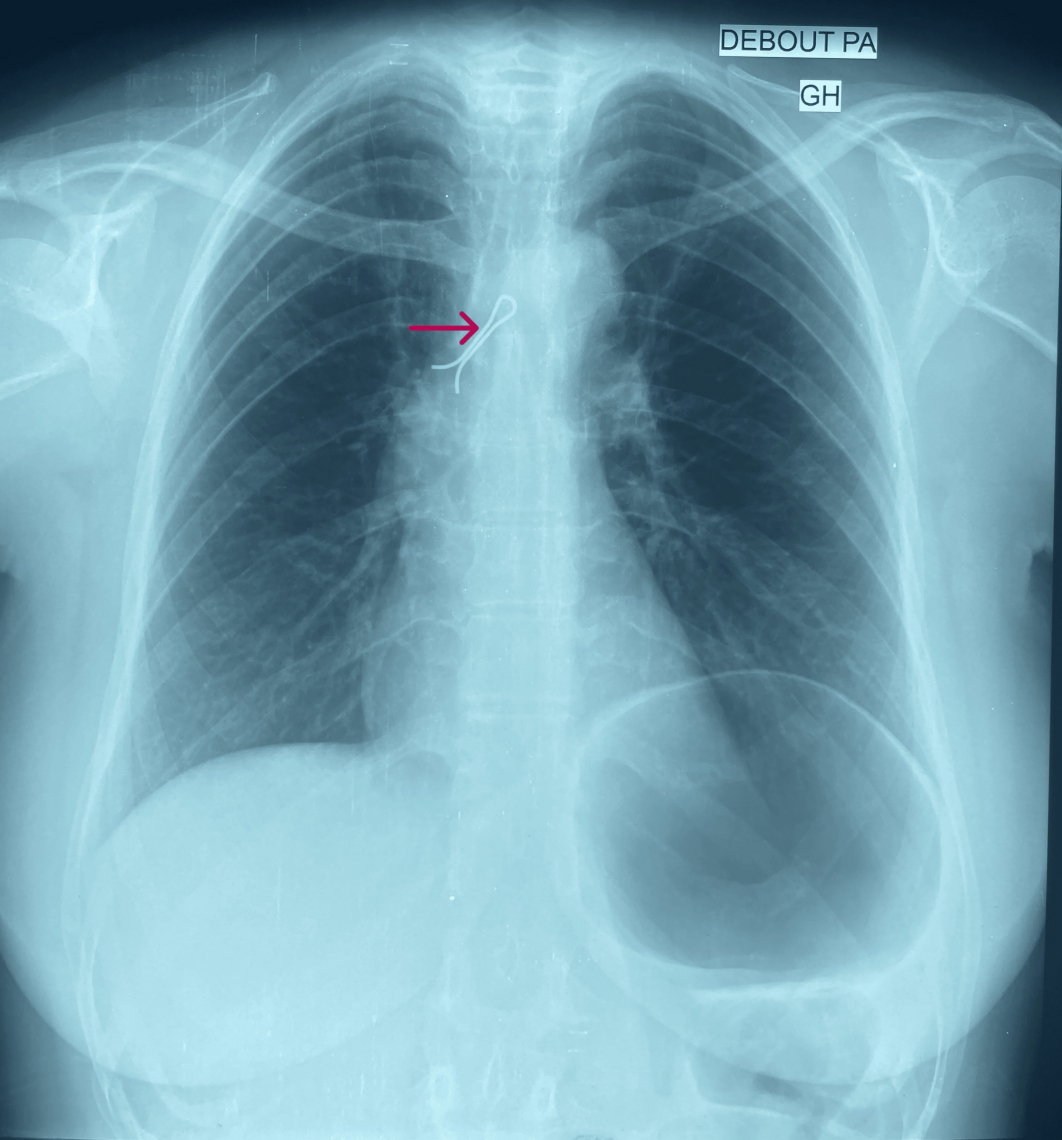
Chest radiograph (PA view) showing a radiopaque shadow (arrow) consistent with hijab pin in right main bronchus PA: posteroanterior

**Figure 2:**
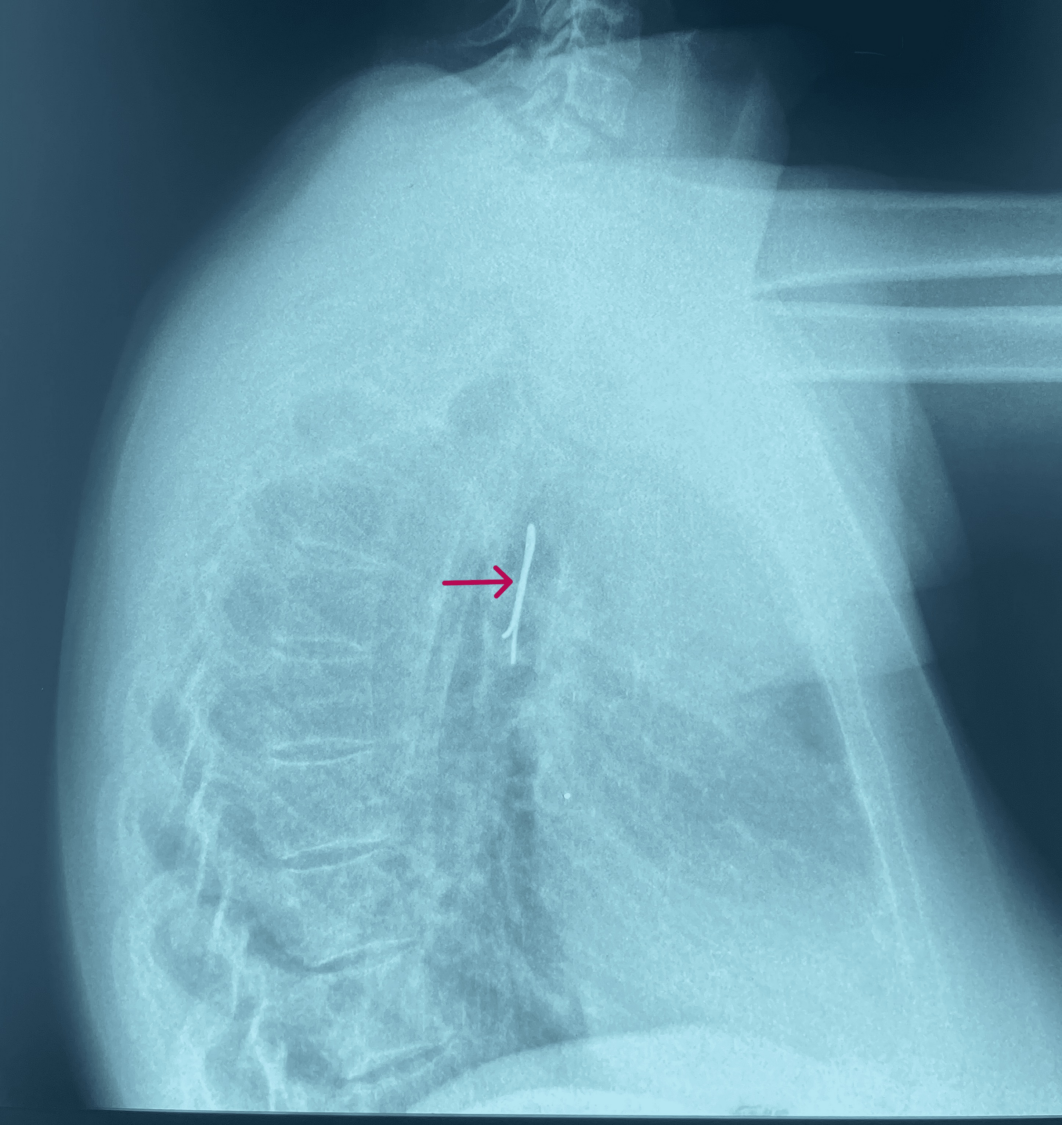
Chest radiograph (lateral view) showing a radiopaque shadow (arrow) consistent with hijab pin in right main bronchus

Chest radiography showed a foreign object in the right main bronchus (Figures 1, 2), and it was decided to urgently attempt removal via flexible bronchoscopy under conscious sedation.

**Figure 3:**
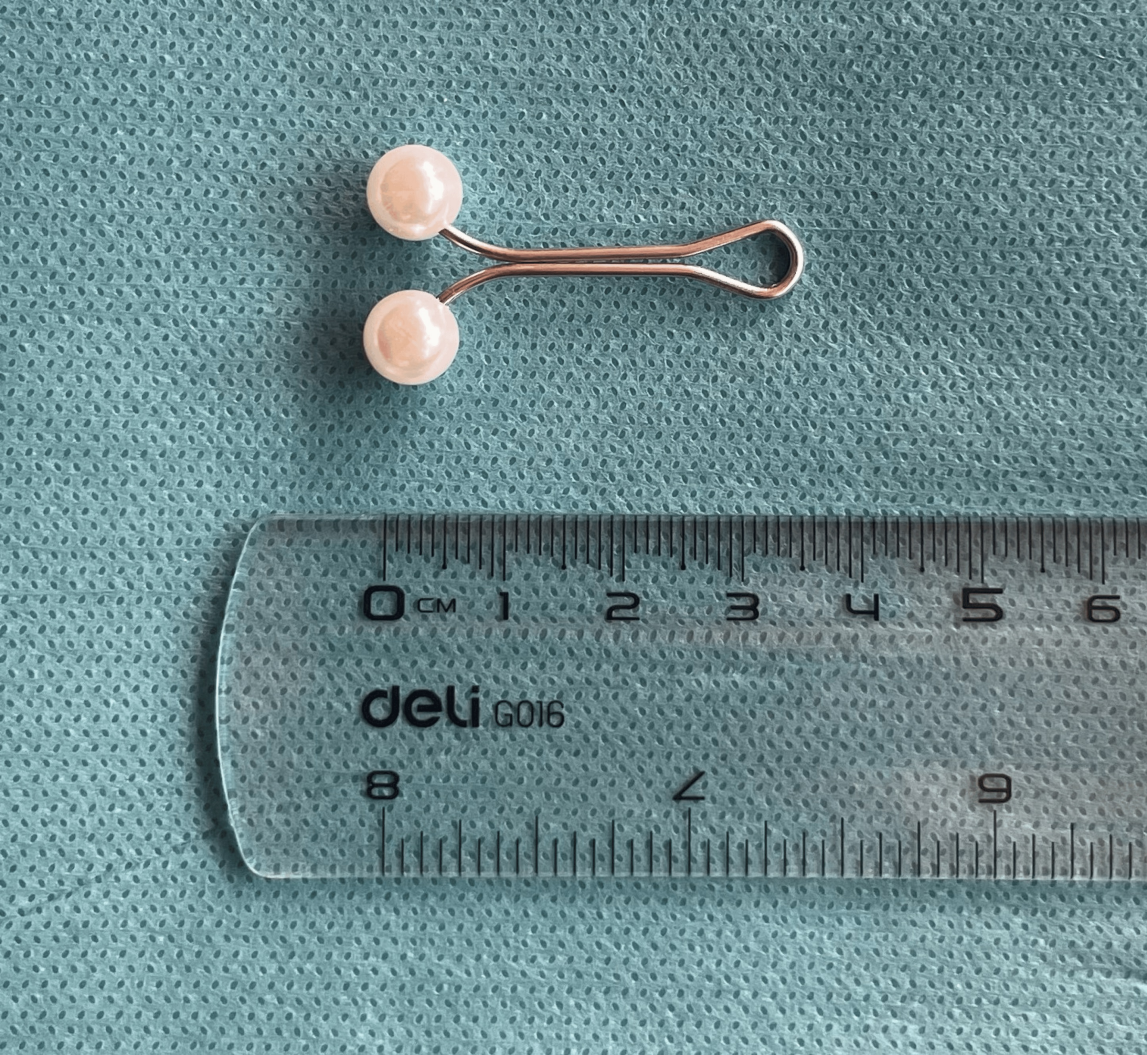
The foreign body (hijab pin) measures approximately 36 mm in length

Before the bronchoscopic procedure, the patient started feeling chest tightness, and fearing an asthma attack, she took two doses of inhaled salbutamol to help alleviate her symptoms. After taking her treatment, the patient had a severe episode of coughing which lasted 1 minute and resulted in coughing up the metallic pin (Figure 3) without the need for bronchoscopic intervention.

The patient was kept under observation for a few hours and discharged home in stable condition.

## Discussion

TFB aspiration is an uncommon occurrence in adults, representing 25% of cases [3]. It can be a life-threatening condition if the object fully obstructs the airway. Inhaled objects in adults are usually lodged in the right bronchus [4] as it is straighter and wider than the left one.

Patient history is important in the evaluation of patients with suspected TFB aspiration. The “penetration syndrome” is usually present - it is a choking episode followed by a persistent cough. It is more common in children than in adults, and the recollection of TFB aspiration in adults is considerably low, averaging about 50% [3].

On examination, wheezing and decreased air entry may be detected on the affected side, but these findings are not specific [3].

In our case, both history and physical exam were highly suggestive of a TFB aspiration as the cause of the patient's acute symptoms.

Radiographic imaging can identify radiopaque objects in 25% of cases and can help localize the foreign body; however, normal X-rays do not rule out the possibility of TFB aspiration as most of them are radiolucent (such as food), and therefore cannot be visible on chest radiograph [5].

CT scan is more performant than radiographs in detecting faintly opaque objects that are surrounded by air [6] and can also detect eventual complications like abscess, empyema, or bronchopulmonary fistula. These complications are more likely to appear the longer the foreign body is not extracted [7]. However, in our patient, the foreign body was a metallic hijab pin which was visible on chest X-ray.

Even though rigid bronchoscopy has been used in the extraction of TFB, there is no gold standard for the removal of TFB in adults [8]. Flexible bronchoscopy is widely being used and can be effective in the diagnosis and extraction of TFB with a high success rate [8-10]. It can be equipped with foreign body forceps for the extraction of large objects. It can be done under conscious sedation reducing the risks associated with general anesthesia during rigid bronchoscopy. It has demonstrated a high failure rate in the extraction of dental prostheses [8]. It may be hazardous for patients who present with respiratory failure [11]; in those patients, rigid bronchoscopy should be preferred and safer to perform.

In our case, before flexible bronchoscopy was performed, the patient coughed up the foreign body (FB) after taking salbutamol.

There was a similar case reported, which resulted in the expulsion of peanut after administration of nebulized salbutamol in a 3-year-old girl [12], and a method of nonendoscopic removal of FB using several doses of beta-2 agonists accompanied by chest physiotherapy was reported [13]. It was effective in 22 of the 24 patients; however, there were two complications: the first happened to a 3-year-old patient, consisting of respiratory and cardiac arrest, and the FB (bean) was extracted by bronchoscopy in pieces; and the second complication happened to a patient of 18 months. It was a respiratory arrest, and the foreign body (peanut) was extracted by direct laryngoscopy after intubation. Both patients survived the life-threatening event. This method is no longer recommended and is considered dangerous [14,15].

Also, in a more recent study [16] that evaluated the administration of nebulized albuterol (beta-2 agonist) + budesonide as a preoperative treatment before bronchoscopy, it was found that this combination significantly reduced bronchoscopic complications such as desaturation and bronchospasm during bronchoscopy and resulted in the spontaneous expulsion of the FB by two patients in the nebulized group with no complications.

## Conclusions

Spontaneous expectoration of TFB aspiration after beta-2 agonist administration is reported in some cases, but some complications were reported after the use of the method and therefore is not recommended. However, it can have some benefits in the immediate preoperative period, with a controlled setting always present for any possible emergency. Removal of TFB aspiration should be performed with either rigid or flexible bronchoscopy, which has been documented to be safe and effective in the removal of TFB aspiration.
